# *Anacardium occidentale* L. (*cajueiro*) in the healing of skin wounds: an experimental study in rats

**DOI:** 10.1590/acb371006

**Published:** 2023-01-06

**Authors:** Dafni Cleia Gaia da Silva, Hanna Machado da Silva, Pedro Pastorini Franco, Tomaz José Aquino Vasconcelos do Carmo, Deivid Ramos dos Santos, Edvaldo Lima Silveira, Ana Carla Godinho Pinto, Marcieni Ataíde de Andrade, Rosa Helena de Figueiredo Chaves

**Affiliations:** 1 Graduate student. Centro Universitário do Estado do Pará – School of Medicine – Belém (PA), Brazil.; 2 Fellow Master degree. Universidade do Estado do Pará – Surgery and Experimental Research – Belém (PA), Brazil.; 3 PhD. Universidade do Estado do Pará – Tropical Medicine – Laboratory for Morphophysiology – Belém (PA), Brazil.; 4 PhD, Associate professor. Faculty of Pharmaceutical Sciences of Universidade Federal do Pará – Experimental Research Group – Belém (PA), Brazil.; 5 PhD, Associate professor. Faculty of Pharmaceutical Sciences of Universidade Federal do Pará – Experimental Research Group – Belém (PA), Brazil.; 6 PhD, Associate professor. Centro Universitário do Estado do Pará – Experimental Research Group – Belém (PA), Brazil.

**Keywords:** Wound Healing, Anacardium, Skin, Rats

## Abstract

**Purpose:**

To analyze the effects of *Anacardium occidentale* Linn on the healing of skin wounds.

**Methods:**

Twenty Wistar male rats were distributed into four groups (with five animals each one): negative control group (NCG), treated with saline solution; cashew tree group (CG), treated with hydroalcoholic extract of the bark of *A. occidentale* Linn; manipulated cashew tree group (MCG), with the ointment of extract of the bark of *A. occidentale* Linn; positive control group (PCG), treated with sunflower oil. All animals were examined daily, observing the macroscopic aspects of the surgical wound. Hematoxylin-eosin staining was used for tissue morphology analysis and Masson’s trichrome for better collagen fiber characterization.

**Results:**

On day 7, the MCG group had the expansion of the surgical wound covered by crust, exceeding the initial limits. On day 21, the wounds were observed to be completely closed and epithelialized in the NCG and CG groups. PCG and MCG still had remnants of crusts on the wound. The NCG was the only one not to present an abscess in histopathological analysis.

**Conclusions:**

There was a prolongation of the healing phase of the animals treated with the extract, and the animals in the NCG showed a better outcome by histological analysis.

## Introduction

The skin is considered the largest organ of the human body^1^^,^ 2 and has primordial functions, such as body defense, maintenance of homeostasis, and thermoregulation^3^^,^ 4. Skin lesion compromises the structure and function of the integument and can be due to trauma, incisions, or intrinsic factors (chronic diseases, such as diabetes or venous vascular insufficiency)^5^. The healing process usually begins soon after the injury and involves events at the cellular, biochemical, and molecular levels to reestablish tissue continuity. The whole process occurs dynamically and can be divided into the phases of inflammation, cell proliferation, and remodeling^6^^-^ 8.

Wound healing is an important issue since there are high financial costs related to treatment and social impact involving the patient^1^^,^ 4 ^,^ 5. In this sense, several alternatives have been described to improve healing, of which the use of medicinal plants stands out in several experimental studies.

Because it has low cost, it is easy to obtain, and it is widely used by the population, medicinal plants are a present ally for the Brazilian popular knowledge, marked by remarkable biodiversity and indigenous culture^1^^,^ 3 ^,^ 6 ^-^ 8. However, the lack of research that isolates the bioactive principles through systematized studies directed at the treatment of wounds is still scarce and hinders the link between scientific and popular knowledge.

In this sense, *Anacardium occidentale* L. stands out, belonging to the Anacardiaceae family (cashew tree). It has antimicrobial^9^^,^ 10, antioxidant^11^, anti-inflammatory^12^, and gastroprotective^13^ effects. One of the explanations for the anti-inflammatory activity of the cashew tree is related to the action of alkylphenols, such as anacardic acids, cardanols, and cardois, which have the antioxidant capacity, reducing the levels of reactive oxygen species and reactive nitrogen species, in addition to decreasing the synthesis of prostaglandins and the activity of inflammatory cells^13^^,^ 14.

Therefore, this work aimed to analyze the effects of hydroalcoholic and ointment extracts of *A. occidentale* Linn bark on the healing of skin wounds in Wistar rats.

## Methods

All experiments were performed following the Brazilian law for the scientific use of animals (Law No. 11,794/08) and the National Institutes of Health (NIH) guide for the care and use of laboratory animals (NIH Publications No. 8,023, revised 1978). The research was approved by the Animal Care and Use Committee of Centro Universitário do Pará (No. 11/2019).

Twenty Wistar male rats (12 weeks old), weighing 350 g, were obtained from Instituto Evandro Chagas. The animals were maintained in individual cages, at 22 °C, under a 12-hour light/dark cycle, and allowed free access to water and standard chow. All surgical procedures and analyses were performed at the Laboratory of Morphophysiology Applied to Health.

### Anesthesia

The animals were anesthetized with 60 mg/kg ketamine, 8 mg/kg xylazine, and 0.03 mg/kg fentanyl intraperitoneally. To verify that the animals were in the anesthetic plane, the caudal, podalic, and vibrissae reflexes were tested, and they were absent.

### Surgical procedure

After anesthesia, all animals were positioned in ventral decubitus and placed on a surgical board (20 × 30 cm). Subsequently, they were submitted to epilation and local antisepsis with polvidine for the creation of the surgical wounds.

A surgical incision was made on the back of the animal with a cold scalpel, measuring 2 × 2 cm and 2-mm deep in the skin of the animals, made after fixed marking with an appropriate stamp. The surgical wound was cleaned with distilled water, simple dripping, and removal of any dirt with the help of sterile gauze. The incision was not sutured, healing by the second intention, and hemostasis was performed by tamponade with gauze.

The animals were distributed into four groups, with five animals each:

- Negative control group (NCG): topically treated with 0.9% saline solution (1 mL), daily, every 12 hours, using a syringe, for 21 days;
- Cashew tree group (CG): topically treated with hydroalcoholic extract of *A. occidentale* Linn the bark 100% (1 mL), daily, every 12 hours, using a syringe without needles, for 21 days;
- Manipulated cashew tree group (MCG): treated with the ointment of extract of *A. occidentale* Linn bark 10%, daily, every 12 hours, with the aid of a cotton swab, for 21 days;
- Positive control group (PCG): treated with sunflower oil (1 mL), daily, every 12 hours, with the aid of a syringe, for 21 days.

All animals were evaluated macroscopically and photographed on days 7, 14, and 21 of the procedure. The animals were euthanized after the 21st day of the procedure.

### Postoperative period and euthanasia

The surgical wounds were not covered. The animals were kept isolated in individual and sterile cages after the surgical procedure, avoiding the occurrence of biting and scratching by another animal at the surgical wound site. Dipyrone was administered subcutaneously at a dose of 30 mg/kg every 12 hours for five days during the postoperative period.

The animals were euthanized by anesthetic overdose with ketamine 180 mg/kg and xylazine 30 mg/kg at the end of the study.

### Extraction

For the extraction method^4^, the dried barks of *A. occidentale* were macerated and crushed, and 471.38 g were placed in 2.7 L of 70% ethanol, at room temperature, with occasional agitation, in a closed container, for seven days, protected from light:

- Hydroalcoholic extract: the maceration process was repeated, changing only the amount of extracting liquid, which became 2.1 L, due to the intumescence of the plant material. Thus, the crude hydroalcoholic extract of the stem barks;
- Pomade: the extract was concentrated in a rotary evaporator, frozen and lyophilized, for dehydration, thus obtaining powder material, which was used for making the ointment. The material was sent to a compounding pharmacy, in which the 10% pomade was prepared with a solid vaseline base.

### Extract phytochemical prospection

The identification of the chemical classes present in *A. occidentale* Linn extract, following Barbosa’s methodology^15^, is based on the observation of color change or precipitate formation, after the addition of a specific reagente (Table 1):

- Saponins test: the extract was dissolved in 30 mL of distilled water, then boiled and filtered. After cooling, the extract was dissolved in 10 test tubes and shaken vigorously for 15 seconds in a closed tube. After standing for 15 minutes, the maximum height of the foam was measured. If all heights were less than 1-cm foam, the index would be greater than 100;
- Test for reducing sugars: the extract was dissolved in 5 mL of distilled water, and 2 mL of FEHLING A reagent, and 2 mL of FEHLING B reagent were added, and heated in a boiling water bath for 5 minutes. The expected result for reducing sugars is the appearance of a brick-red precipitate;
- Tannins and phenols test: the extract was placed in 40 mL of water, the material was boiled for 10 minutes, and water was added to maintain the volume. After cooling, it was filtered and the reactions for hydrolyzable tannins were performed. When positive, they form dark-blue precipitate and green precipitate for condensed tannins;
- Test for flavonoids: the extract was dissolved in 10 mL of methanol. Five drops of concentrated HCl and magnesium scraps were added. The appearance of pink coloration in the solution indicates positive reaction;
- Test for alkaloids: the extract was dissolved in 5-mL of 5% HCl solution. Four 1-mL portions were separated in a test tube, and five drops of the following reagents were added: a) Bouchardat’s reagent, the expected result is a reddish-orange precipitate; b) Dragendorff’s reagent, the expected result is a brick red precipitate; c) Mayer’s reagent, the expected result is a white precipitate;
- Tests for steroids and triterpenes: the extract was dissolved in 10 mL of chloroform, filtered through activated carbon, and the filtrate was transferred to a test tube. Afterwards, 1 mL of acetic anhydride was added and stirred gently, then concentrated H_2_SO_4_ was carefully added. The expected result is an evanescent blue coloration followed by green, indicating the presence of steroids/triterpenoids, respectively;
- Testing for coumarins: the extract was dissolved in 5 mL of diethyl ether and concentrated in a water bath to 0.5 mL. Drops of the solution were applied to a filter paper to form two spots of approximately 1 cm in diameter. To one of them was added one drop of 1N NaOH solution. Afterwards, half of the stain was covered with dark paper, and the other half was exposed to ultraviolet light. The expected result for the presence of coumarins is blue fluorescence in the exposed part of the stain;
- Test for anthraquinones: the extract was dissolved in 5 mL of toluene, and 2 mL of NH4OH solution (10%) was added and stirred gently. The expected result is the appearance of a pink, red, or violet coloration in the aqueous phase due to the presence of anthraquinones.

**Table 1:** Phytochemical evaluation of *Anacardium occidentale* Linn extract.

Secondary metabolites	Phytochemical evaluation	
	CCD	Colorimetric tests
Tannins	+	+++
Alkaloids	+	+
Saponins	+	+
Reducing sugars	+	-
Flavonoids	-	+
Triterpenes	+	-
Coumarins	-	+
Anthraquinones	-	+

CCD: thin layer chromatography; -: no compounds;

+ weakly positive; ++: positive;

+++ strongly positive.

### Macroscopic analysis

All animals were examined daily, observing the macroscopic aspects of the surgical wound (contraction, secretion, signs of inflammation or necrosis). Measurement was performed with millimeter paper on days 7, 14, and 21. The animals were photographed with a digital camera, fixed on a tripod, and kept at the distance of 15 cm from the wound. The diameter values found were tabulated.

### Microscopic analysis

The surgical specimens were removed after euthanasia with a margin of 1 cm of skin around the lesion. The specimens were identified separately and fixed in 10% formalin for histological slides, and numbered according to the number of the animal and subgroup to which they belonged.

Hematoxylin-eosin (HE) staining was used for tissue morphology analysis and Masson’s Trichrome (MT) for better collagen fiber characterization. Subsequently, the specimens were analyzed by optical microscopy by a pathologist, without his prior knowledge of the group to which each sample belonged, to check the various indicators such as vascular proliferation, polymorphonuclear cells, mononuclear cells, fibroblastic proliferation, collagen fibers, and re-epithelialization.

The parameters were graded on scales from 0 to 3, indicating, respectively, samples with absence, scarce, moderate, or accentuated quantity of the variable analyzed^14^.

### Statistical analysis

BioEstat^®^ software 5.4 was used to perform statistical analysis. Analysis of variance (ANOVA) test was used to compare wound length between the groups, and the Kruskal-Wallis’ test to compare the histological results. p < 0.05 was used for significance.

## Results

### Macroscopy

Crust formation was observed in all groups from the third and fourth days after surgery. When comparing the groups, there was a difference in the appearance of the tissue. The CG and MCG had darker and thicker crusts, while the PCG had a thinner and lighter appearing crust.

On day 7, the MCG had the expansion of the surgical wound covered by crust, exceeding the initial limits, while in the CG and PCM there was the maintenance of the lesion area (Table 2). The animals in the MCG and CG exhibited tissue without secretion. The animals in the PCM group presented crust with the presence of exudate and serous aspects with no signs of contamination.

**Table 2:** Wounds in NCG, CG, MCG, and PCG on days 7, 14, and 21 after the operative procedure.

Time	NCG	CG	MCG	PCG
7^th^	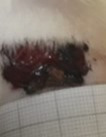	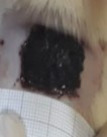	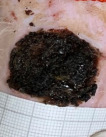	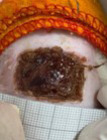
14^th^	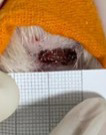	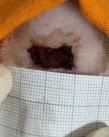	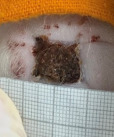	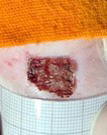
21^rst^	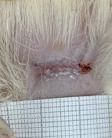	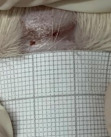	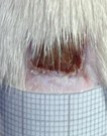	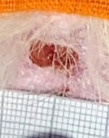

NCG: negative control group; CG: cashew tree group; MCG: manipulated cashew tree group; PCG: positive control group.

On day 14, there was still intense crusting in all groups. The PCM and MCG presented tissue with hyperemia and clots, although the wounds were dry and without exudation.

On day 21, the wounds were observed to be completely closed and epithelialized, and there was complete sloughing off of crusts in the NCG and CG. However, the PCG and MCG still had remnants of crusts on the wound.

In Table 3 it is possible to observe the comparison of wound diameter in the evaluated period.

**Table 3:** Mean values and standard deviation of wound diameter, in centimeters,according to experimental group and healing time.

Group	Diameter						p-value
	Day 7		Day 14		Day 21		
NCG	2	± 0.3	1.5	± 0.1	1.4	± 0.3	0.0056*
CG	2	± 0.1	1.3	± 0.3	1.1	± 0.4	0.0015*
MCG	3.1	± 0.2	1.5	± 0.1	1.2	± 0.2	< 0.0001*
PCG	2.5	± 0.3	1.7	± 0.3	1.1	± 0.2	< 0.0001*

NCG: negative control group; CG: cashew tree group; MCG: manipulated cashew tree group; PCG: positive control group;

* analysis of variance.

Fibroblasts were found in accentuated amounts in all animals. The NCG was the only one that showed scarce fibroblasts in 20% of animals.

Vascular proliferation showed significant difference (p = 0.0467) among the groups. The NCG and GC showed a greater proportion of moderate proliferation, and the NCG and CG showed moderate proliferation in 80%. The MCG and PCG showed a higher proportion of accentuated proliferation and an absence of scarce proliferation (Table 4).

**Table 4:** Microscopic evaluation of fibroblast, vascular proliferation, monomorphonucleates cells,polymorphonucleated cells, reepithelialization, and presence of an abscess.

Variables	GCN		GC		GCM		GCP		p-value
**Number of fibroblasts**									
Scarce	1	20%	0	0%	0	0%	0	0%	> 0.05
Accentuated	4	80%	5	100%	5	100%	5	100%	
**Vascular proliferation**									
Scarce	1	20%	0	0%	0	0%	0	0%	
Moderate	4	80%	4	80%	2	40%	2	40%	0.0467
Accentuated	0	0%	1	20%	3	60%	3	60%	
**Monomorphonucleates cells**									
Scarce	5	100%	4	80%	2	40%	2	40%	0.0390
Moderate	0	0%	1	20%	3	60%	3	60%	
**Polymorphonucleated cells**									
Scarce	5	100%	4	80%	2	40%	2	40%	
Moderate	0	0%	0	0%	0	0%	1	20%	0.0340
Accentuated	0	0%	1	20%	3	60%	2	40%	
**Reepithelialization**									
Moderate	0	0%	2	40%	3	60%	3	60%	> 0.05
Accentuated	5	100%	3	60%	2	40%	2	40%	
**Presence of abscess**									
Presence	0	0%	1	20%	2	40%	3	60%	0.0294
Absence	5	100%	4	80%	3	60%	2	20%	

NCG: negative control group; CG: cashew tree group; MCG: manipulated cashew tree group; PCG: positive control group.

Considering monomorphonucleates, scarce to moderate amounts were found, with statistically significant difference (p = 0.0390) between the groups. The NCG showed 100% of animals with scarce amounts. The GC was close to the NCG (80%). The MCG and PCG showed identical results, with a higher proportion of moderate amounts of monomorphs (60%).

The polymorphnucleates were found in scarce, moderate, and accentuated amounts, with statistically significant difference (p = 0.0390) between the groups. The NCG presented 100% of animals with scarce amounts. The CG was approximate to NCG (80%), but one of the animals showed accentuated amounts. In the MCG, the highest proportion was of animals with marked amounts of polymorphs (60%). The PCG showed equal values (40.0%), in the scarce and accentuated amounts of polymorphs.

Observing reepithelialization classification, moderate to accentuated amounts were found, with no statistical difference (p = 0.0528). The NCG showed all animals with accentuated reepithelization. The GC, GCM, and PCG presented moderate and accentuated amounts.

The presence of abscesses showed statistical difference (p = 0.0294) between the groups. The NCG was the only one not to present an abscess. The group with the highest proportion of animals with the presence of abscesses was the PCG (60%), followed by the GCM (40%) and the GC (20%).

Regarding the presence of collagen fibers, all animals in all groups showed accentuated amounts, with no need to apply statistical tests.

**Figure 1:**
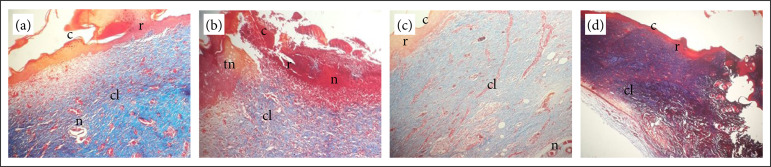
Histopathological aspects of NCG, CG, MCG, and PCG stained with Masson’s trichrome. **(a)** NGC; **(b)** CG; **(c)** MCG magnified at 10x; **(d)** PCG magnified at 5x. It is perceived in 1, 2, and 3 the presence of neovascularization; crust; granulation tissue, composed of collagen, and complete re-epithelialization. In 2, there is granulation tissue with a vascular pattern, a thick crust, neovascularization, and necrotic tissue. In 3, there is vascular pattern granulation tissue, a thick crust, and neovascularization. In 4, it is noted the presence of fibrovascular granulation tissue and complete re-epithelialization.

NCG: negative control group; CG: cashew tree group; MCG: manipulated cashew tree group; PCG: positive control group; c: crust; cl: collagen; n: neovascularization; r: re-epithelialization; tn: necrotic tissue.

**Figure 2:**
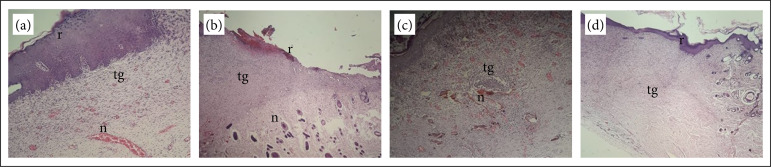
Histopathological aspects of NGC, CG, MCG, and PCG stained with hematoxylin-eosin. **(a)** NGC; **(b)** CG; **(c)** MCG magnified at 10x; **(d)** PCG magnified at 5X. In 1, complete re-epithelialization and neovascularization are observed. In 2, there are granulation tissue, neovascularization, incomplete re-epithelialization (on the left fully re-epithelialized tissue and the right partial re-epithelialization), presence of an ulcer in the central region of the crust, and a crust with hemorrhage. In 3, vascular pattern granulation tissue and re-epithelialization are evidenced. In 4, complete re-epithelialization, granulation tissue, and absence of appendages at the site of healing are observed.

NCG: negative control group; CG: cashew tree group; MCG: manipulated cashew tree group; PCG: positive control group; tg: granulation tissue; n: neovascularization; r: re-epithelialization; tn: necrotic tissue.

By Masson’s trichrome (Fig. 1) stain and HE (Fig. 2), it was possible to observe the variables described in Table 4.

## Discussion

### Macroscopic analysis

The measurement of the area of a wound is an important indicator to assess the evolution of the healing process^16^^-^ 18, and it can be done by observing the time of evolution, depth, vitality of neighboring tissues, presence of secretion, necrosis, among others.

In this study, there was a statistically significant difference when comparing the diameter of the surgical wound, especially in the MCG, which had a more evident reduction between the 7th and 14th day (p < 0.0001), reducing from 3.1 to 1.5 cm in diameter when compared with the control groups. Similar situation was described by Schirato^18^, that showed reduction in wound diameter when animals were treated with polysaccharides from *A. occidentale*.

This occurs because *A. occidentale* acts in the acute phase of inflammation, reducing the formation of edema. It also acts by inhibiting the arachidonic acid cycle, and consequently, decreases cell chemotaxis and the mechanisms of prostaglandins involving a smaller number of fibroblasts^18^. This anti-inflammatory property is due to the anacardic acids and tannins present in the plant^19^, thus reducing the signs of hyperemia and exudate, results found in the CGM group.

Souza^14^ showed that the groups treated with *A. occidentale*-based polysaccharide showed greater wound contraction, progressively reducing the area of the lesion, suggesting greater collagen synthesis. This action of *A. occidentale* can be justified by the increased stimulation of myofibroblasts, synthesizing collagen and elastin^20^^,^ 21, with no additional side effects or adverse reactions during treatment^14^.

### Microscopic analysis

Fibroblasts were found in increased amounts in all animals treated with the extract. Studies show that tannins are important phytoconstituents in the proliferation and activity of fibroblasts^20^, allowing suggest that the extracts used act not only in modulating inflammatory cells, but also accelerating the remodeling process evidenced in the late stages of healing^16^^,^ 22 ^,^ 23.

Moderate amounts of vascular proliferation were found in the CG and NCG, while in the MCG and PCG there was prevalence of accentuated vascular proliferation.

During the proliferative phase, angiogenesis represents the development of new vessels from the vascularity adjacent to the wound. Once blood flow and oxygen partial pressure are reestablished, the stimulus for angiogenesis is inhibited, the newly formed vessels tend to decrease, and it is expected that by day 21 the vascularization is reduced, in an adequate healing process^24^.

This parameter was better achieved in the NCG, which showed 20% of the samples with scarce vascular proliferation, and 80% with moderate proliferation, unlike the other groups, which showed higher percentages of vascularization, similar to what was found in others studies^20^^,^ 25. An improvement in neovascularization was seen in the group treated with hydrogel of *A. occidentale* already from the seventh day^23^ of injury justified by the presence of tannins, a phytoconstituent responsible for wound healing, which promotes increased capillary vessel formation^25^.

In this study, mono- and polymorphonuclear cells were more evident in the groups treated with the extract. The abundant presence of mono- and polymorphonucleated cells are characteristic of the inflammatory phase, an early stage of healing. The abundant presence of these cells at the end of 21 days signals a prolongation of the exudative phase, demonstrating a late healing repair, differing from results found by the previous studies^20^.

This difference can be partly explained because the previously described studies were conducted using oral administration^25^^,^ 26 route without direct contact of the extract on the skin lesion or when applied directly to the wound. Histological collection was performed at only one week of injury, not being evaluated the healing process at later stages^18^^,^ 20 ^,^ 27.

The NCG was the only one not to present abscess. The group with the highest number of animals with abscesses was the PCG (60%), followed by the MCG (40%) and CG (20%).

Because abscesses were not macroscopically visualized during daily evaluations at 21 days, expectant management was performed. According to Schirato^18^, none of the lesions in the groups analyzed showed infection or purulent secretion when submitted to extract of *A. occidentale*, being the only study to report the absence of infectious process in the wound.

The animals in this study were slightly overweight. Although there are studies that show no increased risk of infection of skin lesions in overweight rats^28^, and studies that demonstrate the association^29^, the results showed sugest that this was not the main reason that led to the development of abscesses in the groups, but a factor that increased the possibility, considering in the NCG the presence of abscesses was not identified.

One of the important reasons that may have generated this occurrence may be related to the storage of animals during the postoperative period (non-sterile environment, such as sawdust contaminated with feces and urine of the animals.

## Conclusion

There was prolongation of the healing phase of the animals treated with the extract, and the animals in the NCG showed a better outcome by histological analysis. Further research needs to be done to identify what mechanism is related to the development of abscess and prolongation of healing in rats submitted to treatment with *A. occidentale*.
